# Discovery of Serotransferrin Glycoforms: Novel Markers for Diagnosis of Liver Periductal Fibrosis and Prediction of Cholangiocarcinoma

**DOI:** 10.3390/biom9100538

**Published:** 2019-09-27

**Authors:** Wassana Jamnongkan, Carlito B. Lebrilla, Mariana Barboza, Anchalee Techasen, Watcharin Loilome, Paiboon Sithithaworn, Narong Khuntikeo, Chawalit Pairojkul, Nittaya Chamadol, Raynoo Thanan, Puangrat Yongvanit

**Affiliations:** 1 Department of Biochemistry, Faculty of Medicine, Khon Kaen University, Khon Kaen 40002, Thailand; wassana_jk@hotmail.co.th (W.J.); watclo@kku.ac.th (W.L.); 2 Cholangiocarcinoma Research Institute, Khon Kaen University, Khon Kaen 40002, Thailandnkhuntikeo@gmail.com (N.K.); 3 Department of Chemistry, University of California, Davis, CA 95616, USA; cblebrilla@ucdavis.edu (C.B.L.); mbarboza@ucdavis.edu (M.B.); 4 Faculty of Associated Medical Sciences, Khon Kaen University, Khon Kaen 40002, Thailand; 5 Department of Parasitology, Faculty of Medicine, Khon Kaen University, Khon Kaen 40002, Thailand; paib_sit@kku.ac.th; 6 Department of Surgery, Faculty of Medicine, Khon Kaen University, Khon Kaen 40002, Thailand; 7 Department of Pathology, Faculty of Medicine, Khon Kaen University, Khon Kaen 40002, Thailand; chawalit-pjk2011@hotmail.com; 8 Department of Radiology, Faculty of Medicine, Khon Kaen University, Khon Kaen 40002, Thailand; nittayachamadol@yahoo.com

**Keywords:** biomarkers, serotransferrin, glycobiology, cholangiocarcinoma, periductal fibrosis, mass spectrometry, liver fluke

## Abstract

Cholangiocarcinoma (CCA) caused by chronic liver fluke infection is a major public health problem in Northeast Thailand. Identification of CCA risk groups is urgently needed for the control of CCA in this region. Periductal fibrosis (PDF) induced by chronic inflammation of bile ducts is known as a pre-neoplastic lesion of CCA. We aimed to identify the serum CCA and PDF biomarkers using mass spectrometry (UPLC-ESI-QqQ) with multiple reaction mode (MRM) analysis. Here, serum levels of serotransferrin glycoforms at the glycopeptide level were measured in the sera of CCA (*n* = 100), PDF (*n* = 50), and healthy control (*n* = 100) subjects. The results indicated that serotransferrin peptide levels were generally the same between the control and PDF groups, whereas CCA patients had reduced levels. Moreover, 56 serotransferrin glycoforms were detected, with nine increased in CCA compared to control subjects. Among them, the serum levels of four glycoforms were increased in PDF and CCA patients compared to control subjects. In particular, highly sialylated multi-branched glycans of serotransferrin serum were significantly correlated with poor prognosis and tumor stage in CCA patients. Taken together, these glycoforms could be used as risk biomarkers and prognosis and diagnosis markers of CCA.

## 1. Introduction

Cholangiocarcinoma (CCA) is a slowly progressing tumor, in which patients develop symptoms over a long period of time. Because the majority of CCA cases are clinically silent and not detected in early stages of the disease, CCA is a tumor with a high mortality rate. The known causes of CCA are chronic bile duct inflammatory diseases [1]. In northeast Thailand, where CCA is highly endemic, the major cause of CCA is liver fluke (*Opisthorchis viverrini* (Ov)) infection [2]. This parasite infection induces periductal fibrosis (PDF), which consequently develops into CCA in animal models [3,4]. Thus, PDF is recognized as a CCA risk factor in humans [5]. Since CCA is a major cause of death in Thailand, earlier identification of CCA risk group is urgently needed for better control of CCA in regions where it is endemic. At present, PDF and CCA are usually diagnosed using a combination of several advanced techniques, including abdominal ultrasonography, CT scanning, and tissue biopsy. However, only a limited number of PDF and CCA patients are diagnosed using these techniques due to the high cost and the risk associated with taking samples. Although various tumor markers, such as carcinoembryonic antigen (CEA) and cancer antigen 19-9 (CA-19-9), are used in the diagnosis of CCA, these are not specific for CCA because their levels increase in other cancers and even in benign biliary disorders [6,7]. Additionally, the tumor markers for PDF subjects have not been explored extensively. Therefore, identification and validation of biomarkers that could be used to screen subjects at high risk for PDF and CCA using a simple biochemical method are still needed.

Protein glycosylation, one of the most common post-translational modifications, was proposed as a new source of potential biomarkers for healthy and diseased states [8]. The most common type of protein glycosylation occurs with the addition of specific glycan residues to asparagine (N-linked glycosylation or N-glycans) [9]. Modification of glycosylation influences tumorigenesis by affecting growth, differentiation, metastasis, and immune surveillance. N-glycans are critical in a wide range of biological processes, including protein folding, localization, trafficking, and biological activities, as well as in cell–cell interactions and signal transduction by membrane proteins [10,11,12,13,14]. N-glycans are also involved in many pathological events, such as cancers and congenital disorders of glycosylation [15,16,17].

Serum transferrin (serotransferrin) is the fourth most abundant serum glycoprotein in humans and plays an important role in iron metabolism. In humans, serotransferrin consists of a polypeptide chain of 679 amino acid residues and is synthesized in the liver [18]. It is a bi-lobal protein with N-terminal and C-terminal domains and glycans which attach to the C-terminal domain [19,20]. Serotransferrin has two N-linked glycan chains which mostly exist as complex biantennary types with terminal sialic acids. The protein is fully glycosylated, with these glycans present on two major Asn-linked glycosylation sites, namely, Asn^432^ (Asn-Lys-Ser) and Asn^630^ (Asn-Val-Thr) [21]. Glycosylation is site specific, especially core fucosylation, which occurs only at the Asn^630^ site [22]. Changes in glycosylation of serotransferrin occur in hepatocellular carcinomas, showing an increase in highly branched fucosylated glycans [23].

Glycoproteomics is an important branch of proteomics that identifies carbohydrate types and linkages on polypeptides, and has become a useful tool for biomarker discovery [24,25]. It is now well-established that glycosylation is altered significantly in cancer cells compared to their normal counterparts [26,27,28]. Many potential carbohydrate-related biomarkers (glyco-biomarkers) of various cancers have been discovered and some have been used in clinical practice [29,30]. For example, Her2/neu has been used as the marker for breast cancer, prostate-specific antigen (PSA) for prostate cancer, CA125 for ovarian cancer, and CEA for colorectal, bladder, breast, pancreatic, and lung cancers [24,25]. However, the identification of specific carbohydrate structures on a given glycoprotein is difficult because a large amount of glycoprotein is required for isolation and purification. For better characterization of glycoproteins, a simple method to determine the N-glycans of a target protein is essential.

The structural complexity of glycosylation allows the discovery of novel biomarkers at various levels. The monosaccharides present in a specific glycan can be accurately monitored via mass spectrometry [31,32]. Multiple-reaction monitoring (MRM) on triple quadrupoles (QqQ) mass spectrometry has the potential to be one of the key techniques in this field. The ability to accurately quantitate compounds is an important aspect of mass spectrometry, especially in clinical application. For many years, MRM has been the standard for quantitation in targeted applications, particularly in proteomics [33] and metabolomics [34]. Therefore, MRM methods are suitable for fast, sensitive, and specific quantitative analysis of multiple compounds simultaneously in the presence of other, more abundant compounds in bio-fluids.

In the present study, using Agilent 1290 infinity ultrahigh-performance liquid chromatography (UPLC) system and electrospray ionization (ESI) coupled with an Agilent 6490 triple quadrupoles (QqQ) mass spectrometer (UPLC-ESI-QqQ) for MRM analysis, we determined the N-glycosylation site occupancy of serotransferrin in the sera of the following study subject groups: (i) a CCA risk group (PDF), who were diagnosed with PDF ultrasonographically, with a current or previous history of infection with Ov [5], (ii) CCA patients, and (iii) healthy control subjects. Then, we analyzed the correlations between the degree of glycosylation of serotransferrin and the clinical data of the CCA patients including age, sex, metastasis status, histopathological results, and survival rate.

## 2. Materials and Methods

### 2.1. Human Serum Specimens

Sera of CCA patients (*n* = 100) were obtained from the bio-bank of the Cholangiocarcinoma Research Institute, Khon Kaen University. The research protocols were approved by the Human Research Ethics Committee, Khon Kaen University (#HE571283). Healthy control and PDF sera were obtained from subjects living in the Donchang and Banwa Districts, Khon Kaen Province, Thailand, during an epidemiological survey for Ov infection. Healthy controls (*n* = 100) had no history of Ov infection, as proven by negative results for Ov antibody detection and the fecal egg examination, while PDF subjects (*n* = 50) were Ov-positive by antibody or fecal egg examination and had proven PDF as shown by abdominal ultrasonography performed by a radiologist. Informed consent was obtained from each subject. The protocol of serum sample collection and study design were approved by the Ethics Committee for Human Research, Khon Kaen University (#HE551303). All sera were kept frozen at –20 °C until use. The characteristics of the participants are listed in Table 1.

### 2.2. Tryptic Digestion In Solution

For the reduction of proteins, 20 µL of serotransferrin standard (2 μg/µL; Merck, St. Louis, MO, USA) and 2 µL of serum samples were added to 2 µL of 550 mM dithiothreitol (DTT; Promega, Madison, WI, USA) and incubated at 60 °C in a water bath for 50 min, then alkylated by adding 4 µL of 450 mM iodoacetamide (IAA; Merck) and incubated at room temperature in the dark for 30 min. Then, the solution was digested with trypsin (enzyme to substrate ratio = 1:50) at 37 °C for 16 h. The resulting peptide and glycopeptide samples were cleaned up using a C-18 Zip-Tip column (Agilent Technologies, Santa Clara, CA, USA) prior to mass spectrometry (UPLC-ESI-QqQ).

### 2.3. UPLC-ESI-QqQ analysis

An Agilent Eclipse plus C18 column (RRHD 1.8 μm, 2.1 × 100 mm) was used for UPLC separation. The peptide samples were analyzed using UPLC-ESI-QqQ mass spectrometry (Agilent Technologies). Briefly, the serotransferrin standard sample was diluted serially in nano-pure water prior to injection to obtain a calibration curve for protein quantitation. For each run, 1 μL of sample was injected. Three replicate injections were performed for each serotransferrin standard solutions to evaluate the instrument repeatability. A nano-pure water blank was run after every three-sample run to evaluate potential carry over. To access the stability of the sample preparation during the experiment, we included two serotransferrin standard samples (Merck) before and after running the patient samples.

After injection of 5 μL of serum sample, peptides and glycopeptides were separated using a 10 min binary gradient consisting of 3% acetonitrile and 0.1% formic acid for solvent A and 90% acetonitrile and 0.1% formic acid for solvent B in nano-pure water (*v*/*v*) at a flow rate of 0.5 mL/min. The QqQ instrument was used at dynamic MRM mode, in which transitions were monitored only when the target molecules were eluted. The transitions were monitored. The retention times and collision energies used re summarized in Supplementary Table S1. The MRM results were analyzed using Agilent MassHunter Quantitative Analysis B.5.0 software. The peak area was integrated using quantitation software. The detection limit was defined as a signal-to-noise ratio of ≥3.

MRM transitions were developed on the QqQ-MS, and the instrument parameters were optimized to obtain best sensitivity for the glycopeptides. The peptides and glycopeptides were both quantified using MRM in the same run. The absolute amount of serotransferrin protein was determined using a peptide-calibration curve, while the degree of glycosylation was normalized to the total protein content. Using this strategy, protein abundance of the protein and degree of glycosylation were monitored simultaneously and at the site-specific level, as described by Hong et al. (2015) [35].

### 2.4. Statistical Analysis

Data were expressed as graphs and were analyzed using GraphPad Prism 5 (GraphPad Software, San Diego, CA, USA) Statistical analyses were performed using IBM SPSS version 19.0 software (IBM Cooperation, Armonk, NY, USA). Survival curves were calculated according to the method of Kaplan and Meier. A *p*-value of <0.05 was considered to be statistically significant. Identification of glycopeptides and glycan patterns was performed using in-house software designed by Barboza et al. (2012) [36]. Glycopeptide composition was assigned on the basis of the exact mass and fragmentation patterns. The diagnostic performances of the serotransferrin peptides and glycopeptides were evaluated using receiver operating characteristic (ROC) curve analysis. The area under the ROC curve (AUC) was determined using 95% Confidence Interval (CI). Data were presented as mean ± SD.

## 3. Results

### 3.1. Absolute Quantiytation of Serotransferrin in Serum

**Figure 1:**
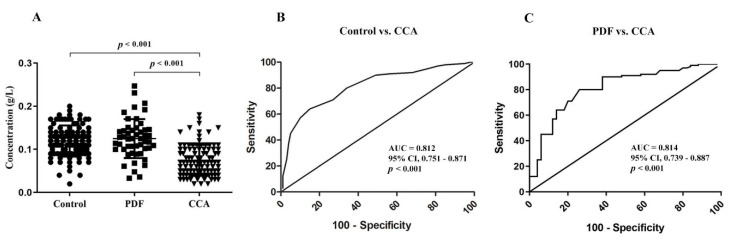
Distribution of serotransferrin peptides in serum. (**A**) Absolute quantitation of serotransferrin peptides were determined using the multiple reaction mode (MRM) method. Data are represented as mean ± SD. Receiver operating characteristic (ROC) curves in subjects with CCA (*n* = 100) compared with the control group (*n* = 100) are represented in (**B**). ROC curves in patients with PDF (*n* = 50) compared to CCA (**C**) are constructed. The area under ROC curve (AUC) and statistical comparisons are indicated.

**Figure 2:**
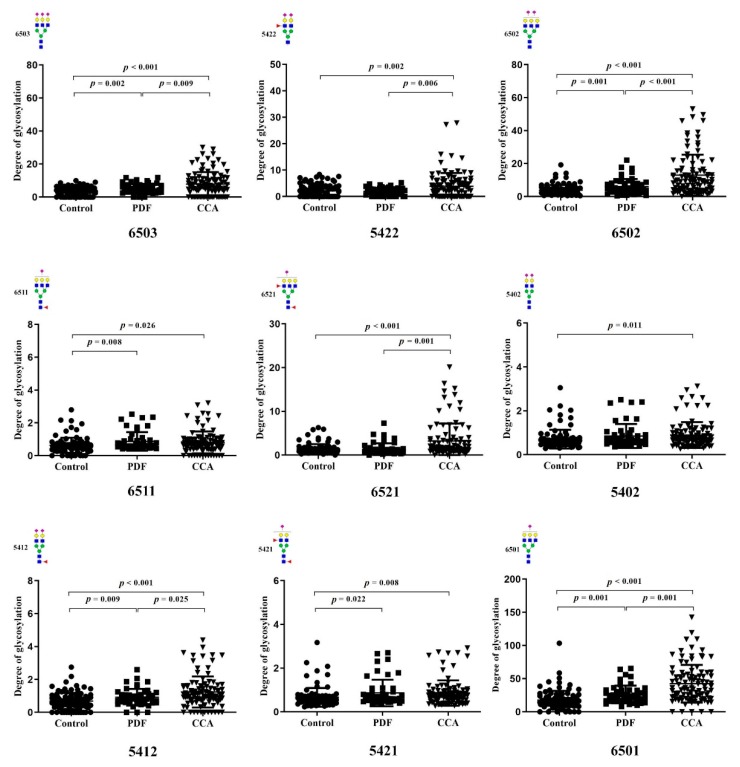
Serum levels (mean ± SD) of altered serotransferrin glycofoms at Asn^432^ and Asn^630^ residues in healthy control (*n* = 100), PDF (*n* = 50) and CCA (*n* = 100) subjects. The 6503, 5422, 6502, 6511, and 6521 glycoforms were detected at the Asn^432^ residue. The 5402, 5412, 5421, and 6501 glycoforms were detected at the Asn^630^ residue. *P*-values were calculated using Student’s t-test. The glycan forms are represented in glycan composition numbers of hexose, hexNAc, fucose, and N-acetyl neuraminic acid, respectively. The pictures of glycoform structures represent of N-acetylglucosamine (blue square), mannose (green circle), galactose (yellow circle), N-acetyl neuraminic acid (pink diamond), and fucose (red triangle).

Serotransferrin levels of each investigated group are presented in Figure 1A as scatter plots. CCA patients had significantly lower serotransferrin levels than the healthy control and PDF groups. The areas under the ROC curves (AUC) (Figure 2B,C) between the control group, the PDF group, and the CCA group were 0.812 (95% CI, 0.751–0.871; *p* < 0.001) and 0.814 (95% CI, 0.739–0.887; *p* < 0.001), respectively. 

### 3.2. Glycopeptides of Serotransferrin Standard

The total MRM chromatograms of N-linked glycopeptides in a tryptic digest of serotransferrin standard at Asn432 and Asn630 are shown in Figure S1. The glycan forms were represented in glycan composition numbers of hexose (Hex), hexNAc, fucose (Fuc), and N-acetyl neuraminic acid (NeuAc), respectively. Two sizes of tryptic digested peptides of Asn^432^, including (i) ^421^CGLVPVLAENYNK^433^ and (ii) ^421^CGLVPVLAENYNKSDNCEDTPEAGYFAIAVVK^452^, were observed. The Hex6HexNAc5NeuAc1 (6501), Hex6HexNAc5NeuAc2 (6502), Hex5HexNAc4 Fuc2 (5420), Hex5HexNAc4NeuAc1 (5401), Hex5HexNAc4Fuc2NeuAc1 (5421), Hex5HexNAc4NeuAc2 (5402), Hex4HexNAc3NeuAc1 (4301), and Hex5HexNAc4Fuc1NeuAc2 (5412) were detected at Asn^432^ of the ^421^CGLVPVLAENYNK^433^ peptide. Also, Hex5HexNAc4NeuAc2 (5402), Hex5HexNAc4Fuc2NeuAc1 (5421), Hex5HexNAc4Fuc1NeuAc1 (5411), Hex5HexNAc4Fuc2NeuAc2 (5422), Hex4HexNAc3 (4300), and Hex6HexNAc5NeuAc2 (6502) glycoforms were detected at Asn^432^ of the ^421^CGLVPVLAENYNKSDNCEDTPEAGYFAIAVVK^452^ peptide. The Hex5HexNAc4Fuc2NeuAc2 (5422), Hex4HexNAc3 (4300), Hex4HexNAc3NeuAc1 (4301), Hex5HexNAc4NeuAc1 (5401), Hex5HexNAc4Fuc2 (5420), Hex5HexNAc4Fuc2NeuAc1 (5421), Hex5HexNAc4NeuAc2 (5402), Hex5HexNAc4Fuc1NeuAc2 (5412), Hex6HexNAc5NeuAc1 (6501), and Hex5HexNAc4Fuc1NeuAc1 (5411) glycoforms were detected at the Asn^630^ glycosite. Most of the glycoforms of N-glycosylation at Asn^432^ and Asn^630^ detected in this study have been previously reported in earlier works [37,38] using nano liquid chromatography (LC)-chip/quadrupole time-of-flight mass spectrometry (MS) analysis and 360 MHz proton magnetic resonance spectroscopy. 

### 3.3. Identification of Altered Glycosylation at Asn^432^ and Asn^630^ of Serotransferrin in CCA

Nine glycoforms were significantly higher in CCA sera compared with control sera. The glycan moieties overexpressed in the sera of CCA patients were Hex6HexNAc5NeuAc3 (6503), Hex5HexNAc4Fuc2NeuAc2 (5422), Hex6HexNAc5NeuAc2 (6502), Hex6HexNAc5Fuc1NeuAc1 (6511), and Hex6HexNAc5Fuc2NeuAc1 (6521) at the Asn^432^ residue, and Hex5HexNAc4NeuAc2 (5402), Hex5HexNAc4Fuc1NeuAc2 (5412), Hex5HexNAc4Fuc2NeuAc1 (5421), and Hex6HexNAc5NeuAc1 (6501) at the Asn^630^ residue. Figure 2 shows the serum levels of the altered glycoforms in the control, PDF, and CCA groups. The degree of glycosylations (mean ± SD) in each glycoform are shown in Table S2. Table 2 shows the AUC values of each glycan for the control, PDF, and CCA groups. The ROC curves are shown in Figure S2–S10. Among them, the serum levels of 6503, 6502, 6501, and 5412 were significantly different among the control, PDF, and CCA subjects.

### 3.4. The Association of Altered Glycoforms of Serotransferrin and Clinicopathological Data of CCA Patients

**Figure 3:**
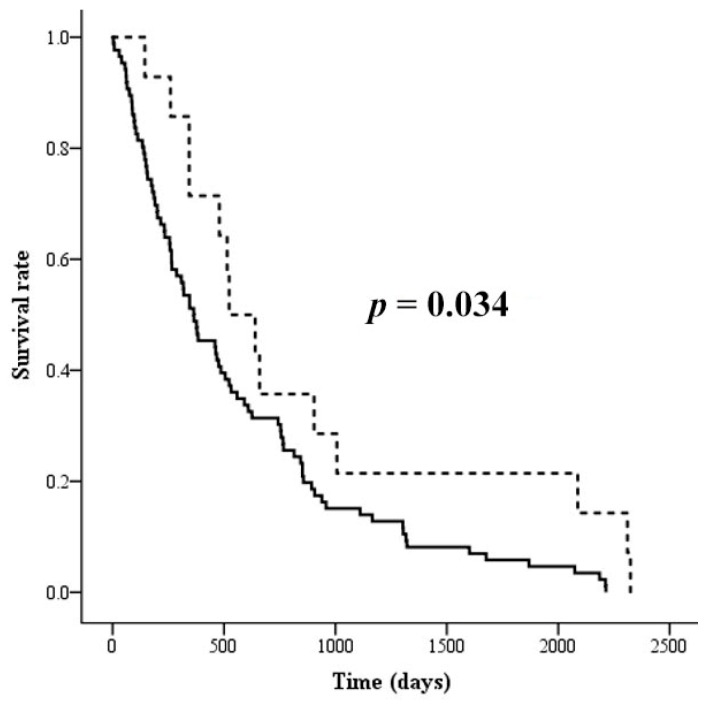
Survival curves calculated for levels of the 6503, 6502, and 6501 serotransferrin glycoforms in CCA sera, according to Kaplan–Meier with a log-rank test. The solid line represents CCA patients with high expression of 2 or more of the 3 glycoforms (*n* = 86). The dashed line represents CCA patients with high expression of 1 or none of the 3 glycoforms (*n* = 14).

Levels of serotransferrin peptides and glycopeptides in the sera of CCA patients were analyzed in accordance with clinicopathological data, including sex, age, histology type, survival rate, and metastasis stage of patients. There was no correlation between any of the glycoform levels and the clinical data, including sex, age, histology type, and metastasis stage of the patients, as shown in Tables S3 and S4. High 6501 glycoform levels were significantly correlated with the tumor stage (*p* = 0.034), as shown in Table S3. CCA patients with high expression of 2 or more of the glycoforms 6403, 6502, and 6501 showed a significant correlation with poor prognosis (*p* = 0.034) (Figure 3). 

## 4. Discussion

Our study revealed that the serotransferrin protein levels of CCA patients were significantly lower than that of the control and PDF groups. Serotransferrin is a negative acute-phase protein involved with physiological changes responsible for various stimuli, including tissue injury, infection, and immunological disorders, and it is also reduced in chronic inflammation and malignant growth [39,40]. Serum serotransferrin levels have been reported to decrease in ovarian and other gynecological cancer patients [41,42] and during inflammation [43]. After chemotherapy, levels of serotransferrin increased or remained constant in ovarian cancer patients [41]. Therefore, the decrease in serotransferrin in the sera of CCA patients may be due to the physiological response of stress caused by CCA development.

Liver fluke infection is a major etiology of CCA in Thailand. This parasite infection induces chronic inflammation and tissue injury, resulting in PDF formation and CCA development [3,4]. PDF was also identified as a CCA risk factor in humans and can be detected by abdominal ultrasonography [5]. It was reported that anti-inflammatory and antioxidant agents, such as curcumin, anthocyanin, and xanthohumol, can be used for protection of PDF formation and CCA development in animal models [44,45,46]. Therefore, CCA can be prevented by not eating raw cyprinid fish and by treatment with anthelmintics in case of parasitic infection to avoid PDF development. Once PDF does occur, expert radiologists are required for diagnosis. 

In the present study, dynamic MRM using a QqQ-MS instrument was applied to identify differential glycosylation patterns of serotransferrin in the sera of CCA patients. Six glycoforms were shown to be significantly increased in the sera of PDF subjects compared to control group. These were the 6503, 6502, 6511, and 6521 glycoforms at the Asn^432^ residue and the 5402, 5421, and 6501 glycoforms at the Asn^630^ residue. Additionally, the 6503, 6511, 5422, 6502, and 6521 glycoforms at the Asn^432^ residue and the 5402, 5412, 5421, and 6501 glycoforms at the Asn^630^ residue were significantly increased in CCA patients compared to control subjects. Among them, the serum levels of four glycoforms (6503, 6502, 6501, and 5412) were significantly increased in CCA compared to PDF subjects and in PDF compared to control subjects. Therefore, although the serum levels of serotransferrin protein itself are reduced in CCA patients, the serum levels of particular serotransferrin glycoforms could be used as PDF biomarkers and CCA risk biomarker. 

Glycan composition and its relevance to the biological activities of human serotransferrin have been extensively studied in relation to hepatocellular carcinoma (HCC), carbohydrate deficiency syndrome type II, and pregnancy [47,48,49]. CCA and HCC are both liver cancers and share similar signs and symptoms. An increase in the 5200, 7400, 6300, and 6310 serotransferrin glycoforms in the sera of HCC was previously reported by Yamashita et al. [49]. These glycoforms lack sialic acid, therefore their increase may be due to the decrease in sialytransferase activity or the increase in sialidase activity in HCC. In contrast, in this study, the sialylated glycoforms (6503, 6502, 6501, and 5412) were significantly increased in the sera of CCA patients compared to healthy subjects. Therefore, these serotransferrin glycoforms may be used as biomarkers to differentiate between CCA and HCC patients.

Serotransferrin glycoforms have been reported to induce anti-apoptotic properties on hematopoietic cells and lymphocytes [50]. Notably, CCA patients with high expression of two or more of three (6503, 6502, and 6501) glycoforms had a higher likelihood of a poor prognosis and CCA patients with high expression of the 6501 glycoform had a higher likelihood of a high tumor stage. These three glycoforms are sialylated glycans without fucosylation. Sialylated N-glycans generally play essential roles in cancer cell survival, drug resistance, and cancer cell metastasis [51]. Moreover, the total sialic acid in the sera of CCA patients was significantly higher compared to patients with benign hepatobiliary diseases and healthy subjects [52]. The increase in total sialic acid in the sera of CCA was significantly correlated with the clinical data, including serum MUC5AC mucin, alkaline phosphatase, and CA19-9, and the proportions of white blood cells and neutrophils [53]. Sialyltransferases were found to be up-regulated and induced tumor progression in many cancer types [54]. In conclusion, sialytransferases may be up-regulated or sialidase may be down-regulated in CCA and the increase in sialylated serotransferrin glycoforms may play a critical role in carcinogenesis, leading to tumor promotion and progression with worse clinical outcomes, such as poor prognosis.

## 5. Conclusions

The present study indicated that alterations of serotransferrin glycoforms could be used as potential risk biomarkers and diagnostic or prognostic biomarkers in CCA. Since serotransferrin is an abundant glycoprotein in human serum, analysis of its glycoforms could increase sensitivity and specificity in CCA risk group identification. Additional studies regarding the serotransferrin protein and its site-specific glycosylation profiles could allow further elaboration on the characteristics of biological functionality and causality of altered glycosylation in CCA. Further development of a specific detection system for the altered glycoforms should be explored.

## Figures and Tables

**Table 1:** Characteristics of study subjects.

Variable	Control (*n* = 100)	^#^ PDF (*n* = 50)	^$^ CCA(*n* = 100)
Age (Years)			
<56	57	29	41
>56	43	21	59
Gender			
Male	42	22	46
Female	58	28	54
Histological grading			
Papillary	-	-	55
Tubular	-	-	45
Metastasis stage	-	-	48
Non-metastasis	-	-	52

^#^ PDF represents periductal fibrosis subjects, and ^$^ CCA represents cholangiocarcinoma pateints

**Table 2:** The differentiation powers (^#^ AUC values) of serotransferrin glycoforms.

Group	^$^ Glycoform								
	6503	5422	6502	6511	6521	5402	5412	5421	6501
Control vs. PDF	0.608 *	0.511	0.651 **	0.632 **	0.578	0.596 *	0.653 **	0.596	0.653 **
Control vs. CCA	0.681 ***	0.593 *	0.792 ***	0.610 **	0.696 ***	0.637 **	0.710 ***	0.637 **	0.710 ***
PDF vs. CCA	0.604 *	0.595	0.682 ***	0.500	0.735 ***	0.544	0.609 *	0.539	0.685 **

^#^*P*-values are represented as * *p* < 0.05, ** *p* < 0.01, and *** *p* < 0.001. ^$^ The glycoforms are represented in glycan composition numbers of hexose, hexNAc, fucose, and N-acetyl neuraminic acid, respectively.

## Supplementary Materials

The following are available online at https://www.mdpi.com/2218-273X/9/10/538/s1. Table S1: Dynamic MRM transitions used to monitor glycopeptides; Table S2: Degree of glycosylation (mean ± SD) of the altered glycoforms in control, PDF and CCA; Table S3: Association between levels of serotransferrin peptides and glycopeptides in the sera of CCA patients and clinicopathological data; Table S4: Association between levels of 6503, 6502, and 6501 serotransferrin glycopeptides in the sera of CCA patients and clinicopathological data; Figure S1.:Total MRM chromatogram of serotransferrin standard and its glycopeptides; Figure S2: ROC curve analysis of the Hex6HexNAc5NeuAc3 (6503) glycoform in the sera of control (*n* = 100), PDF (*n* = 50), and CCA (*n* = 100) subjects; Figure S3: ROC curve analysis of the Hex5HexNAc4Fuc2NeuAc2 (5422) glycoform in the sera of control (*n* = 100), PDF (*n* = 50), and CCA (*n* = 100) subjects; Figure S4: ROC curve analysis of the Hex6HexNAc5NeuAc2 (6502) glycoform in the sera of control (*n* = 100), PDF (*n* = 50), and CCA (*n* = 100) subjects; Figure S5: ROC curve analysis of the Hex6HexNAc5Fuc1NeuAc1 (6511) glycoform in the sera of control (*n* = 100), PDF (*n* = 50), and CCA (*n* = 100) subjects; Figure S6: ROC curve analysis of the Hex6HexNAc5Fuc2NeuAc1 (6521) glycoform in the sera of control (*n* = 100), PDF (*n* = 50), and CCA (*n* = 100) subjects; Figure S7: ROC curve analysis of the Hex5HexNAc4NeuAc2 (5402) glycoform in the sera of control (*n* = 100), PDF (*n* = 50), and CCA (*n* = 100) subjects; Figure S8: ROC curve analysis of the Hex5HexNAc4Fuc1NeuAc2 (5412) glycoform in the sera of control (*n* = 100), PDF (*n* = 50), and CCA (*n* = 100) subjects; Figure S9: ROC curve analysis of the Hex5HexNAc4Fuc2NeuAc1 (5421) glycoform in the sera of control (*n* = 100), PDF (*n* = 50), and CCA (*n* = 100) subjects; Figure S10: ROC curve analysis of the Hex6HexNAc5NeuAc1 (6501) glycoform in the sera of control (*n* = 100), PDF (*n* = 50), and CCA (*n* = 100) subjects.
