# Supplementary material for: Discovery of Serotransferrin Glycoforms: Novel Markers for Diagnosis of Liver Periductal Fibrosis and Prediction of Cholangiocarcinoma
Source: Biomolecules. 2019 Sep 27;9(10):538. doi: 10.3390/biom9100538 (PMC6843847; doi:10.3390/biom9100538)
Supplement: Supplementary file 1 [file biomolecules-09-00538-s001.pdf]

Supplementary Tables and Figures

Table 1. Dynamic multiple reaction mode (MRM) transitions used to monitor glycopeptides**.**

Glycosite	Glycofom		Precursor ion (m/z)	Product ion (m/z)	Collision energy (eV)	Retention time (min)
	#Component	$Number				
Asn[21–433]	H4N3	4300	912.1	204.0	14	6.6
	H4N3S1	4301	1009.1	204.0	16	6.6
	H4N4S1	4401	1076.8	274.1	17	7.8
	H5N4	5400	1033.8	366.1	16	6.6
	H5N4S1	5401	1130.8	366.1	18	6.4
	H5N4S2	5402	1227.8	366.1	20	6.6
	H5N4F1	5410	1082.5	366.1	17	6.6
	H5N4F1S1	5411	1179.5	274.1	19	7.6
	H5N4F1S2	5412	1276.5	366.1	21	6.6
	H5N4F2	5420	1131.2	366.1	18	6.4
	H5N4F2S1	5421	1228.2	274.1	20	6.6
	H5N4F2S2	5422	994.2	366.1	16	6.6
	H6N5S1	6501	1252.5	366.1	20	6.3
	H6N5S2	6502	1012.4	274.1	16	6.5
	H6N5S3	6503	1085.2	274.1	17	6.7
	H6N5F1S1	6511	967.2	274.1	15	7.7
	H6N5F2	6520	1252.9	366.1	20	6.4
	H6N5F2S1	6521	1012.7	366.1	16	6.6
	H6N5F2S2	6522	1085.4	274.1	17	6.7
Asn[421–452]	H4N3	4300	1201.0	366.1	19	7.6
	H4N3F1	4310	1273.8	366.1	21	8.0
	H4N4S1	4401	1059.9	366.1	17	8.0
	H5N4	5400	1292.3	366.1	21	8.0
	H5N4S1	5401	1092.3	366.1	17	8.0
	H5N4S2	5402	1150.5	366.1	18	6.6
	H5N4F1	5401	1063.3	366.1	17	8.0
	H5N4F1S1	5411	1121.5	366.1	18	7.5
	H5N4F1S2	5412	1179.7	274.1	19	7.5
	H5N4F2	5420	1092.5	366.1	17	8.0
	H5N4F2S1	5421	1150.7	366.1	18	6.5
	H5N4F2S2	5422	1208.9	274.1	19	7.5
	H6N5S1	6501	1165.3	366.1	19	8.0
	H6N5S2	6502	1223.5	274.1	20	8.0

Glycosite	Glycofom		Precursor ion (m/z)	Product ion (m/z)	Collision energy (eV)	Retention time (min)
	#Component	$Number				
	H6N5S2	6502	1223.5	274.1	20	8.0
	H6N5S3	6503	1281.7	366.1	21	8.0
	H6N5F1S1	6511	1194.5	366.1	19	7.5
	H6N5F1S2	6512	1252.7	366.1	20	6.3
	H6N5F2S1	6521	1223.7	366.1	20	8.1
	H6N5F2S2	6522	1281.9	274.1	21	6.7
Asn$^{622–642}$	H4N3	4300	1258.2	366.1	20	7.6
	H4N3S1	4301	1016.7	274.1	16	7.6
	H4N4S1	4401	1067.4	366.1	17	7.6
	H5N4	5400	1035.2	366.1	16	7.6
	H5N4S1	5401	1108.0	366.1	18	7.6
	H5N4S2	5402	1180.7	274.1	19	7.5
	H5N4F1	5410	1071.7	366.1	17	7.6
	H5N4F1S1	5411	1144.5	366.1	18	10.4
	H5N4F1S2	5412	1217.2	274.1	20	7.5
	H5N4F2	5420	1108.2	366.1	18	7.3
	H5N4F2S1	5421	1181.0	274.1	19	7.6
	H5N4F2S2	5422	1253.8	366.1	20	6.6
	H6N5S1	6501	1199.2	366.1	19	7.6
	H6N5S2	6502	1272.0	274.1	21	5.9
	H6N5S3	6503	1076.0	274.1	17	7.8
	H6N5	6500	1126.5	366.1	18	7.9
	H6N5F1S1	6511	1235.8	366.1	20	6.6
	H6N5F1S3	6513	1105.2	366.1	18	7.9
	Peptide Tf		489.8	735.4	11	4.8

Glycan components are hexose (H), hexNAc (N), fucose (F), and N-acetyl neuraminic acid (S). $ The glycan forms are represented in glycan composition numbers of hexose, hexNAc, fucose, and N-acetyl neuraminic acid, respectively.

Table S2. Degree of glycosylation (mean ± SD) of the altered glycoforms in control, periductal fibrosis (PDF), and cholangiocarcinoma (CCA) subjects.

$Glycoform	Degree of glycosylation (mean ± SD)		
	Control	PDF	CCA
**6503**	3.6 ± 2.5	5.0 ± 2.8	7.9 ± 7.3
**5422**	2.1 ± 2.3	1.9 ± 1.4	3.9 ± 5.0
**6502**	3.8 ± 3.1	5.9 ± 4.7	13.0 ± 12.5
**6511**	0.6 ± 0.5	0.9 ± 0.6	0.8 ± 0.7
**6521**	1.5 ± 1.2	1.3 ± 1.4	3.2 ± 4.0
**5402**	0.7 ± 0.4	0.9 ± 0.5	0.9 ± 0.6
**5412**	0.7 ± 0.5	0.9 ± 0.5	1.2 ± 0.9
**5421**	0.7 ± 0.4	0.9 ± 0.6	0.9 ± 0.6
**6501**	17.6 ± 14.0	26.2 ± 13.3	42.4 ± 28.3

$ The glycan forms are represented in glycan composition numbers of hexose, hexNAc, fucose, and N-acetyl neuraminic acid, respectively.

Table 3. Association between levels of serotransferrin peptides and glycopeptides in the sera of CCA patients and clinicopathological data.

Peptide and glycoforms	$Level (n)	Age		Gender		Histological grading		Metastasis stage		#CCA stage				
		<56	≥56	Male	Female	Papillary	Tubular	Negative	Positive	0	1	2	3	4
**Serotransferrin peptide**	Low (91)	38	53	42	49	49	42	47	44	4	6	16	21	31
	High (9)	3	6	4	5	6	3	5	4	2	0	0	2	3
	P-value		0.733		1.000		0.505		1.000					0.136
6503	Low (29)	14	15	13	16	17	12	14	15	4	2	4	8	10
	High (71)	27	44	33	38	38	33	38	33	2	4	12	15	24
	P-value		0.377		1.000		0.665		0.665					0.438
5422	Low (44)	18	26	24	20	28	16	25	19	3	4	9	9	15
	High (56)	23	33	22	34	27	29	27	29	3	2	7	14	19
	P-value		1.000		0.159		0.157		0.426					0.701
6502	Low (26)	12	14	11	15	14	12	15	11	2	2	5	8	6
	High (74)	29	45	35	39	41	33	37	37	4	4	11	15	28
	P-value		0.644		0.819		1.000		0.649					0.626
6511	Low (38)	18	20	19	19	21	17	18	20	1	2	6	7	20
	High (62)	23	39	27	35	34	28	34	28	5	4	10	16	14
	P-value		0.403		0.543		1.000		0.538					0.131
6521	Low (45)	21	24	17	28	21	24	21	24	3	2	6	12	11
	High (55)	20	35	29	26	34	21	31	24	3	4	10	11	23
	P-value		0.315		0.161		0.159		0.422					0.618
5402	Low (45)	21	24	21	24	23	22	24	21	2	2	9	11	17
	High (55)	20	35	25	30	32	23	38	27	4	4	7	12	17
	P-value		0.315		0.074		0.547		0.843					0.823
5412	Low (22)	12	10	10	12	12	10	11	11	2	0	3	6	9
	High (78)	29	49	36	42	43	35	41	37	4	6	13	17	25
	P-value		0.219		0.068		1.000		1.000					0.624
5421	Low (42)	20	22	21	21	22	20	23	19	2	2	8	11	14
	High (58)	21	37	25	33	33	25	29	29	4	4	8	12	20
	P-value		0.305		0.546		0.688		0.688					0.905
6501	Low (16)	4	12	8	8	9	7	6	10	2	0	4	0	10
	High (84)	37	47	38	46	46	38	46	38	4	6	12	23	24
	P-value		0.178		0.789		1.000		0.277					0.034

$The mean values of glycan levels in control subjects were used as the cut-off between high and low glycan levels. #85 cases of CCA had complete staging data.

Table 4. Association between levels of 6503, 6502, and 6501 serotransferrin glycopeptides in the sera of CCA patients and clinicopathological data.

Glycoforms	CCA group (n)	Age		Gender		Histological grading		Metastasis stage		[#]CCA stage				
		<56	≥56	Male	Female	Papillary	Tubular	Negative	Positive	0	1	2	3	4
**6503, 6502, 6501**	[*]High >2 glycoforms (86)	37	49	40	46	46	40	44	42	3	5	13	22	28
	[$]Other (14)	4	10	6	8	9	5	8	6	3	1	3	1	6
	P-value		0.388		1.000		0.567		0.777					0.114

[*]CCA patients with high expression of 2 or more of the 3 glycoforms (n = 86). [$]CCA patients with high expression of 1 or none of the 3 glycoforms (n = 14). [#]85 cases of CCA had complete staging data.

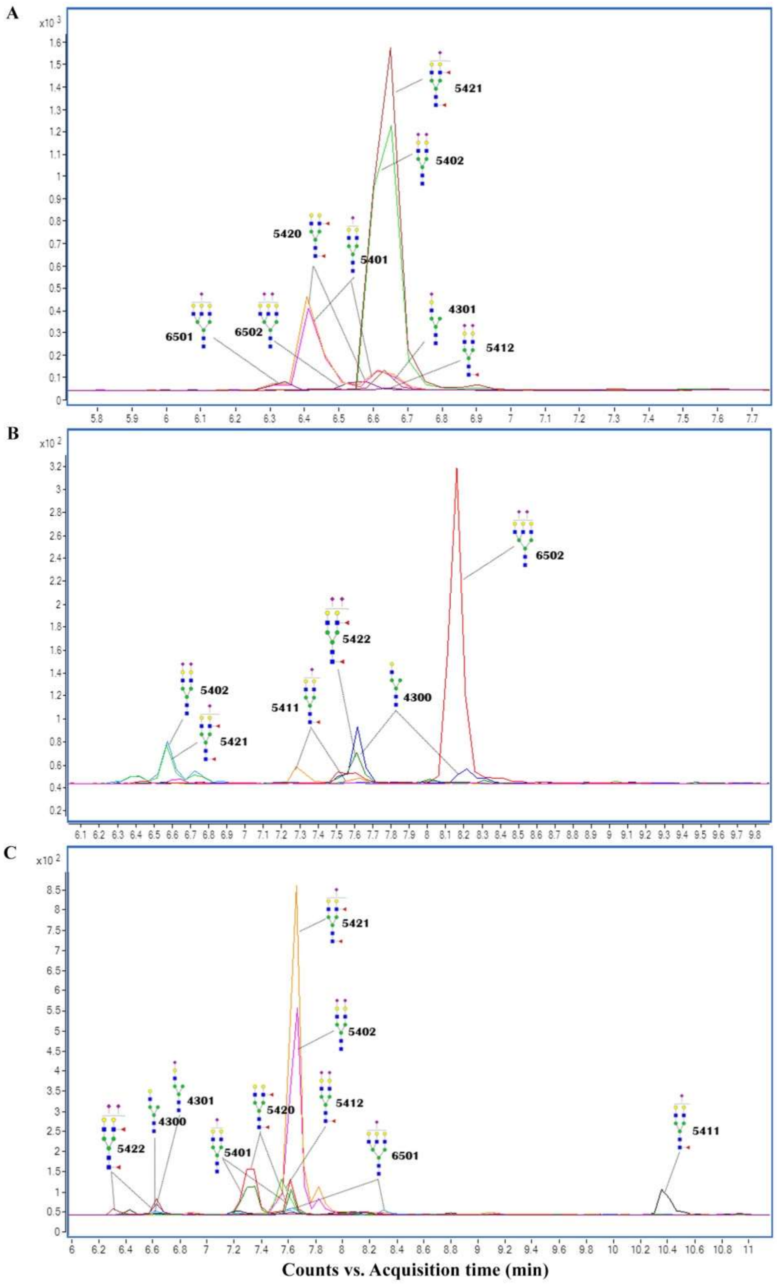

Figure 1. Total MRM chromatogram of standard serotransferrin and its glycopeptides. Chromatograms of glycopeptides in a tryptic digest of standard serotransferrin at (**A**) Asn[432] (CGLVPVLAENYNK), (**B**) Asn[432] (CGLVPVLAENYNKSDNCEDTPEAGYFAIAVVK), and (**C**) Asn[630] (QQQHLFGSNVTDCSGNFCLFR).

6503 glycoform

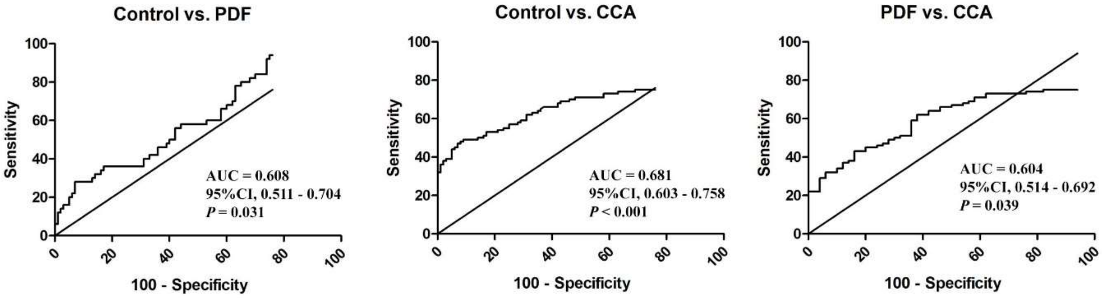

Figure S2. Receiver operating characteristic (ROC) curve analysis of the Hex6HexNAc5NeuAc3 (6503) glycoform in the sera of control (n = 100), PDF (n = 50), and CCA (n = 100) subjects. The ROC curves of the 6503 glycoform of subjects with PDF and CCA compared with the control group are represented in (**A**) and (**B**), respectively. ROC curves of patients with PDF and CCA (**C**) are constructed. Area under the ROC curve (AUC) and statistic comparisons are indicated.

5422 glycoform

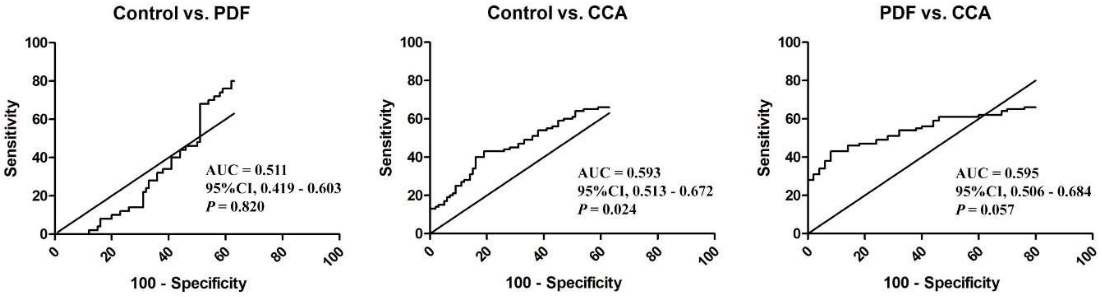

Figure 3. Receiver operating characteristic (ROC) curve analysis of the Hex5HexNAc4Fuc2NeuAc2 (5422) glycoform in the sera of control (n = 100), PDF (n = 50), and CCA (n = 100) subjects. The ROC curves of the 5422 glycoform of subjects with PDF and CCA compared with the control group are represented in (**A**) and (**B**), respectively. ROC curves of patients with PDF and CCA (**C**) are constructed. Area under the ROC curve (AUC) and statistic comparisons are indicated.

6502 glycoform

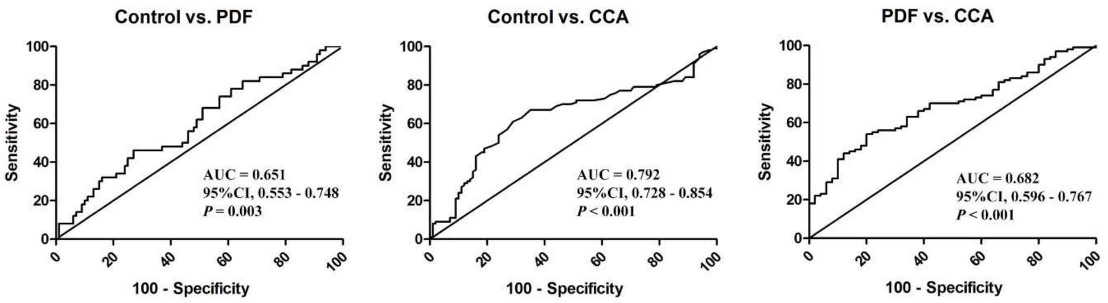

Figure S4. Receiver operating characteristic (ROC) curve analysis of the Hex6HexNAc5NeuAc2 (6502) glycoform in the sera of control (n = 100), PDF (n = 50), and CCA (n = 100) subjects. The ROC curves of the 6502 glycoform of subjects with PDF and CCA compared with the control group are represented in (**A**) and (**B**), respectively. ROC curves of patients with PDF and CCA (**C**) are constructed. Area under the ROC curve (AUC) and statistic comparisons are indicated.

6511 glycoform

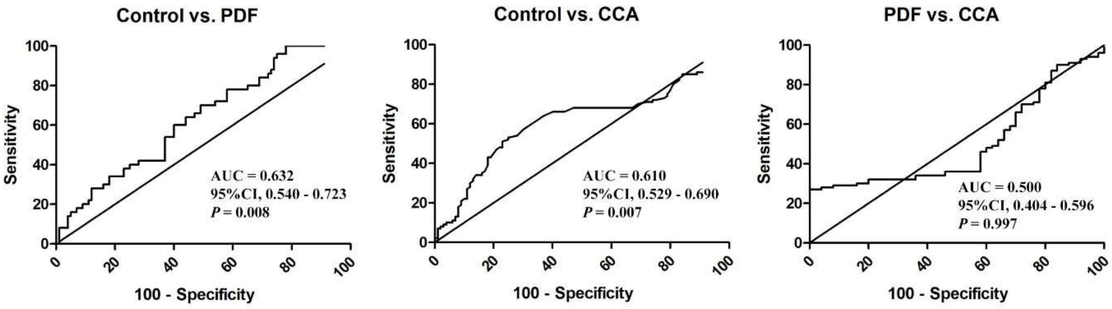

Figure 5. Receiver operating characteristic (ROC) curve analysis of the Hex6HexNAc5Fuc1NeuAc1 (6511) glycoform in the sera of control (n = 100), PDF (n = 50), and CCA (n = 100) subjects. The ROC curves of the 6511 glycoform of subjects with PDF and CCA compared with the control group are represented in (**A**) and (**B**), respectively. ROC curves of patients with PDF and CCA (**C**) are constructed. Area under the ROC curve (AUC) and statistic comparisons are indicated.

6521 glycoform

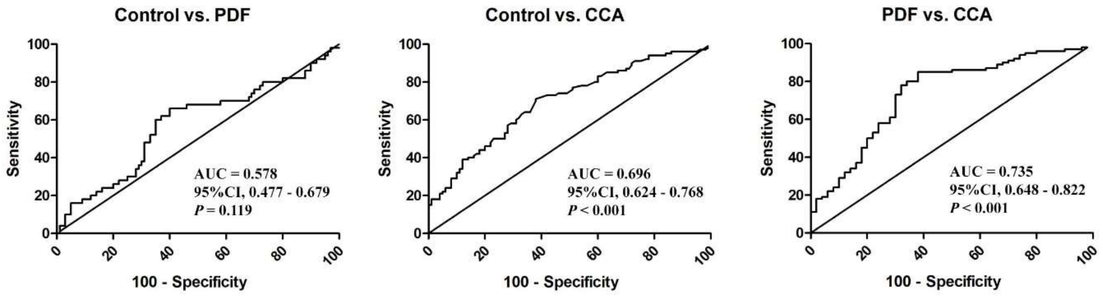

Figure S6. Receiver operating characteristic (ROC) curves analysis of the Hex6HexNAc5Fuc2NeuAc1 (6521) glycoform in the serum of control (n = 100), PDF (n = 50), and CCA (n = 100) subjects. The ROC curves of the 6521 glycoform in the sera of subjects with PDF and CCA compared with the control group are represented in (**A**) and (**B**), respectively. ROC curves of patients with PDF and CCA (**C**) are constructed. The area under the ROC curve (AUC) and statistic comparisons are indicated.

5402 glycoform

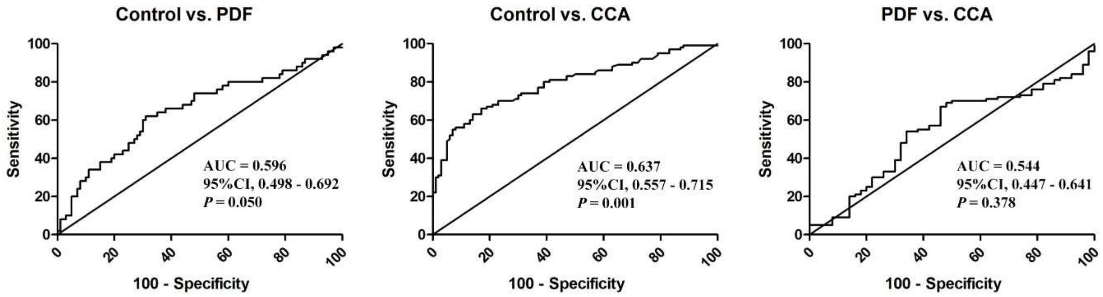

Figure 7. Receiver operating characteristic (ROC) curve analysis of the Hex5HexNAc4NeuAc2 (5402) glycoform in the sera of control (n = 100), PDF (n = 50), and CCA (n = 100) subjects. The ROC curves of the 5402 glycoform in subjects with PDF and CCA compared with the control group are represented in (**A**) and (**B**), respectively. ROC curves of patients with PDF and CCA (**C**) are constructed. Area under the ROC curve (AUC) and statistic comparisons are indicated.

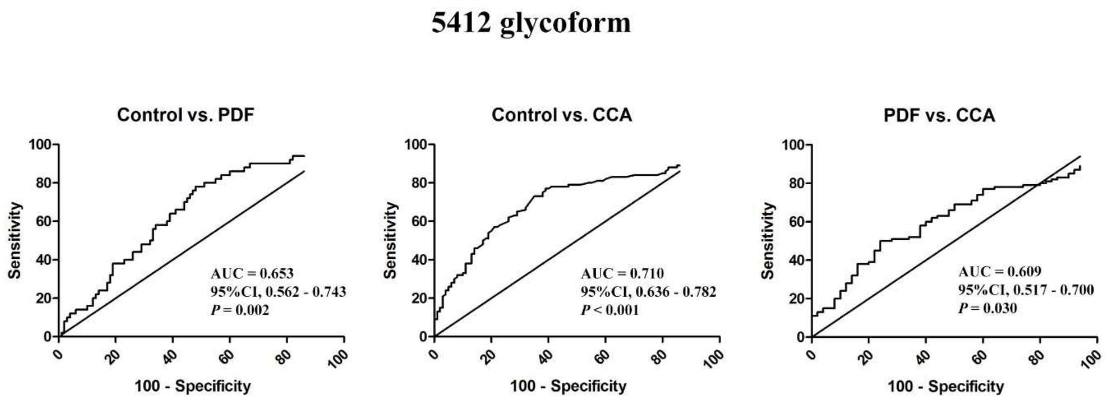

Figure 8. Receiver operating characteristic (ROC) curve analysis of the Hex5HexNAc4Fuc1NeuAc2 (5412) glycoform in the sera of control (n = 100), PDF (n = 50), and CCA (n = 100) subjects. The ROC curves of the glycoform of subjects with PDF and CCA compared with the control group are represented in (**A**) and (**B**), respectively. ROC curves of patients with PDF and CCA (**C**) are constructed. The area under the ROC curve (AUC) and statistic comparisons are indicated.

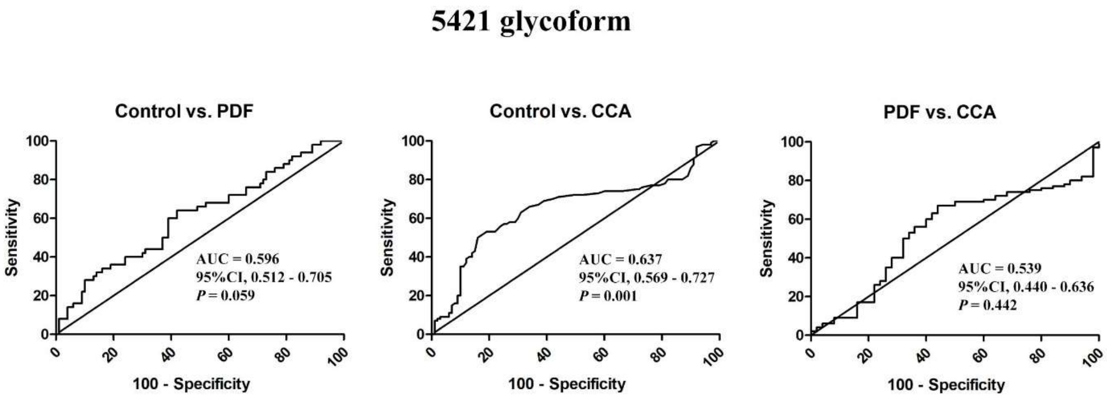

Figure 9. Receiver operating characteristic (ROC) curve analysis of the Hex5HexNAc4Fuc2NeuAc1 (5421) glycoform in the sera of control (n = 100), PDF (n = 50), and CCA (n = 100) subjects. The ROC curves of the 5421 glycoform of subjects with PDF and CCA compared with the control group are represented in (**A**) and (**B**), respectively. ROC curves of patients with PDF and CCA (**C**) are constructed. Area under the ROC curve (AUC) and statistic comparisons are indicated.

6501 glycoform

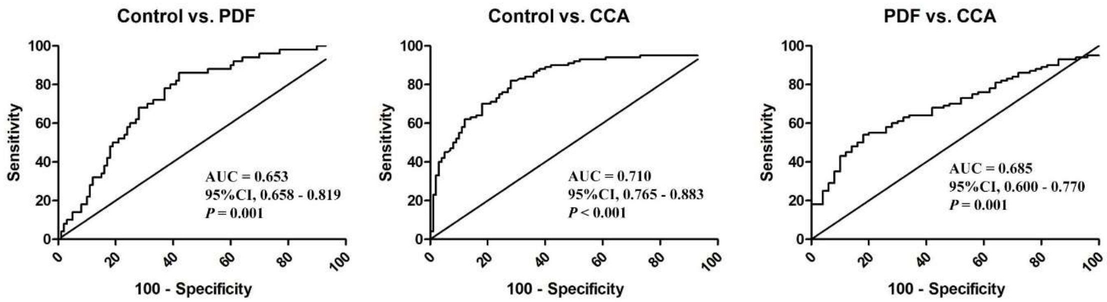

Figure S10. Receiver operating characteristic (ROC) curve analysis of the Hex6HexNAc5NeuAc1 (6501) glycoform in the sera of control (n = 100), PDF (n = 50), and CCA (n = 100) subjects. The ROC curves of the 6501 glycoform of subjects with PDF and CCA compared with the control group are represented in (**A**) and (**B**), respectively. ROC curves of patients with PDF and CCA (**C**) are constructed. Area under the ROC curve (AUC) and statistic comparisons are indicated.
